# When Eating Right, Is Measured Wrong! A Validation and Critical Examination of the ORTO-15 Questionnaire in German

**DOI:** 10.1371/journal.pone.0135772

**Published:** 2015-08-17

**Authors:** Benjamin Missbach, Barbara Hinterbuchinger, Verena Dreiseitl, Silvia Zellhofer, Carina Kurz, Jürgen König

**Affiliations:** 1 Department of Nutritional Sciences, University of Vienna, Vienna, Austria; 2 Department of Psychiatry and Psychotherapy, Division of Social Psychiatry, Medical University of Vienna, Vienna, Austria; Kyoto University, JAPAN

## Abstract

The characteristic trait of individuals developing a pathological obsession and preoccupation with healthy foods and a restrictive and avoidant eating behavior is described as orthorexia nervosa (ON). For ON, neither universal diagnosis criteria nor valid tools for large-scale epidemiologic assessment are available in the literature. The aim of the current study is to analyze the psychometric properties of a translated German version of the ORTO-15 questionnaire. The German version of the ORTO-15, a eating behavior and dieting habits questionnaire were completed by 1029 German-speaking participants (74.6% female) aged between 19 and 70 years (M = 31.21 ± 10.43 years). Our results showed that after confirmatory factor analysis, the best fitting model of the original version is a single-factor structure (9-item shortened version: ORTO-9-GE). The final model showed only moderate internal consistency (Cronbach’s alpha = .67), even after omitting 40% of the original question. A total of 69.1% participants showed orthorectic tendencies. Orthorectic tendencies are associated with special eating behavior features (dieting frequency, vegetarian and vegan diet). Education level did not influence ON tendency and nutritional students did not show higher ON tendency compared to students from other disciplines. This study is the first attempt to translate and to evaluate the psychometric properties of a German version of the ORTO-15 questionnaire. The ORTO-9-GE questionnaire, however, is only a mediocre tool for assessing orthorectic tendencies in individuals and shows moderate reliability and internal consistency. Our research suggests, that future studies are needed to provide more reliable and valid assessment tools to investigate orthorexia nervosa.

## Introduction

There is a thin line between eating right and healthy and a pathological preoccupation with healthy foods. This appears paradox at first, because public health nutrition policies’ primary strategy is to promote healthy dietary choices and eating right to decrease diet-related pathologies like overweight and obesity [1]. In contrast, there are mounting reports from eating disorder professionals who find themselves confronted with individuals who are pathologically preoccupied with healthy eating: a condition called orthorexia nervosa (ON) [2]. ON was first framed by Bratman and Knight [3] in the late 90’s, describing eating behavior associated with behavioral and psychiatric traits. Individuals with ON are typically concerned about food quality rather than food quantity. Nevertheless, there is still a lack of valid instruments for ON [4]. The aim of this study is to provide a validated measurement tool for ON in German language, based on the original (English) ORTO-15 questionnaire [5] and further contribute to our understanding of mediators for orthorectic eating and the identification of groups at risk to develop ON.

### Orthorexia Nervosa Symptoms and Diagnostics

ON is an eating-related condition with obsessive eating directed at healthy foods. The healthfulness of foods can vary depending on individual’s preferences. ON can, in extreme cases, lead to a pathological preoccupation with pure and unprocessed foods and stringent eating plans, combined with significant psychopathological overlappings with anorexia nervosa (AN) and obsessive-compulsive disorders (OCD) [6]. Transgressing self-imposed dietary rules often lead to intense feelings of anxiety, guilt and shame followed by more stringent dietary restriction leading to a vicious cycle [7].

Different from common eating-related disorders, individuals with ON do not fear to gain weight and have clear, rationalized rules related to food intake [4]. Additionally, eating according to a fixed schedule and spending a lot of time to prepare meals [8–10], and unrealistic food beliefs are very prominent among individuals with ON [10]. Social isolation as a consequence of a constant daily domination of healthy eating and reduced stress by eating good and proper foods accompanied by spiritual feelings about foods have been reported [8].

At present, ON is not classified as a formal eating disorder neither by DSM-5 [11], nor by ICD-10 criteria [12]. Grading of ON is still a matter of debate and under current DSM-5 criteria, individuals with ON characteristics may best be classified in the broad category of Avoidant/Restrictive Food Intake Disorder (ARFID) [11]. Several medical consequences are described, which are very similar to other known eating disorders. For instance a shortage of essential nutrients, malnutrition, starvation and weight loss have been reported [13, 14].

### Assessment, Epidemiology and Moderators for Orthorexia Nervosa

To monitor the prevalence of ON and to investigate various subtypes of the condition, thorough assessment tools are needed. Previous investigations report several inconsistencies about the validity and internal reliability of commonly used questionnaires [15]. To date, two instruments were developed to identify individuals with ON: the 10-Item Bratman Scale [3] and the ORTO-15 questionnaire [5]. While the 10-Item Bratman Scale was widely disregarded by the scientific community, several language and item adaptations of the ORTO-15 questionnaires were developed (Polish, Hungarian, Turkish). The only adapted version showing good internal reliability is the Hungarian adaptation (11 items), Cronbach’s alpha = .82. The Polish version (9 items) and the Turkish adaptation (11 items) showed only weak internal reliability of Cronbach’s alpha = .64, respectively Cronbach’s alpha = .62, while in the original work by Donini, Marsili [5], no Cronbach’s alpha was reported. All translated versions of the original ORTO-15 questionnaire deleted various items to increase validity and internal reliability of the original questionnaire [10, 16, 17]. There is no valid instrument to measure ON in German language.

Due to the weakness in assessing ON, there is only limited epidemiological data available on the prevalence of ON and no data from cross-sectional surveys on representative community samples. Donini, Marsili [5] reported a prevalence of 6.9% within different population groups, but these rates should be interpreted with caution because they are not based on a representative study sample.

There are many gaps in the literature concerning potential moderators and risk factors for ON [4]. To fully understand ON, it is important to assess and understand possible moderators for this condition. Hence, there are conflicting results for moderators of ON reported in the literature. As such, high orthorectic tendencies are reported for different population groups (e.g. health professionals [18] and performance artists [16]). Conflicting results are reported whether men or women are more susceptible for ON [5, 13, 19, 20] and if education level mediates ON tendencies [16, 21]. It is also argued that a cultural adaptation for ON is necessary because symptoms of ON may vary across different countries and population groups [10].

### Study Aims

The aims of the current study are threefold. First, we describe the adaptation process of the original ORTO-15 into a German version of the questionnaire; (ii) we investigate the psychometric properties of the translated version via Confirmatory Factor Analysis by applying the instrument on a large and heterogeneous study sample and (iii) we analyse the relationship between socioeconomic, eating and dieting behavior with orthorectic eating tendencies in our study sample.

## Materials and Methods

### Sample

In total, 1538 participants commenced the online survey, while 323 failed to complete the whole questionnaire battery (78.9% completion rate), leaving 1215 participants. 140 participants were excluded because they failed to complete the ORTO-15 questionnaire. Participants with diagnosed diet-related diseases (diabetes mellitus type I and II, Crohn’s disease, celiac disease, gastritis) in which eating behavior has to be adapted for medical reasons were excluded (46 participants) (see S1 Fig for flow chart).

In total, 1029 questionnaires were eligible for further data analysis. To get a more homogenous sample for the descriptive analysis, cut-off thresholds were applied for variables age and BMI (cut-off < 99.9 of the CI). Our study sample consists of 768 women and 261 men (74.6% vs. 25.4%). The mean age of the respondents was 31.21 ± 10.43 years (34.32 ± 11.64 for males; 30.16 ± 9.78 for females). BMI (based on self reported body weight and height) ranged between 15.24 kg/m^2^ and 54.21 kg/m^2^, with a mean weight of 23.33 kg/m^2^ ± 4.37 kg/m^2^. Most participants were students (N = 377, 36.6%), with more than half of the students enrolled in courses in nutritional sciences or dietetics (N = 208, 20.2%), other health-related courses (e.g. medicine, aging-management, nursery; N = 12, 1.2%), and from other fields (e.g. marketing, business, food sciences; N = 157, 15.3%). Employed participants included following professions: business office jobs (N = 157, 15.3%), health professionals (N = 106, 10.3%), social work (N = 80, 7.8%), tourism (N = 72, 6.9%), flight attendants (N = 69, 6.7%), food sector (N = 69, 6.7%), other professions (e.g. informatics, architecture, law; N = 263, 25.6%).

### Procedure

Participants were recruited via online advertisement (social media, email distribution lists) and we collected data online. Participants received the link to a survey called ‘Eating Behavior and Health Aspects’ and as an incentive, four prices were raffled among four participants who completed the entire set of questions (total value 200€). Participants completed an informed consent form, entered sociodemographic information, completed a German translation of the ORTO-15 questionnaire and an additional questionnaire battery. The questionnaire battery consisted of questions about lifestyle and eating behavior habits, the ORTO-15 questionnaire and additional ON related questions. The study protocol was approved by the University of Vienna Ethics Committee (reference number: 00115). Participants were informed that they could withdraw their participation at any time during the online questionnaire.

### Measures

#### Orthorexia Nervosa: ORTO-15

The ORTO-15 questionnaire is a self-report 15-item measure with a 4-point Likert scale (Table 1). It is originally constructed based on a combination of the Minnesota Multiphasic Inventory [22] and the Bratman test [3] to measure the interrelationship between cognitive-rational (items 1, 5, 6, 11, 12, 14), clinical (items 3,7, 8, 9, 15) and emotional aspects (items 2, 4, 10, 13) of eating behavior [5]. The ORTO-15 questionnaire assesses beliefs about attitudes covering food selection (item 4), the extent to which food concerns influence daily life (item 7), the perceived effects of eating healthy food (item 12) and habits of food consumption (item 15). Lower overall scores refer to more ON components (increased ON tendency). Donini, Marsili [5] report sensitivity, specificity, and predictive validity values for the ORTO-15 using an original cut-off < 40 (maximum score = 60) in an Italian adult sample. Other studies used e.g. median split to define individuals with or without ON tendencies [19].

We developed a German version of the ORTO-15 questionnaire by using a multistep translation method as suggested by Sousa and Rojjanasrirat [23]. Briefly, two professional translators (no health care background) translated the ORTO-15 questionnaire into German without adding words or introducing new expressions. Both translations were merged in accordance with the authors (BM; VD; SZ, CK). One clinical psychologist and Author 2 (BH) created the final version of the questionnaire. Afterwards, this version was again back translated into English language by a professional translator and the last version was compared to the final German version within the project team (BM; VD; SZ, CK). At this stage of process, we checked for possible differences in meaning of both versions, all remarks were integrated into the final version of the questionnaire.

**Table 1:** ORTO-15 full text in English and the translated German version.

Item	German Translation	English original
*1*	*Achten Sie beim Essen auf den Kaloriengehalt der Lebensmittel*?	*When eating*, *do you pay attention to the calories of the food*?
*2*	*Fühlen Sie sich beim Lebensmitteleinkauf überfordert*?	*When you go in a food shop do you feel confused*?
3	Haben Sie sich in den letzten 3 Monaten beim Gedanken an LM Sorgen gemacht?	In the last 3 months, did the thought of food worry you?
4	Bestimmt die Sorge um Ihren Gesundheitszustand Ihre Essensauswahl?	Are your eating choices conditioned by your worry about your health status?
5	Ist Ihnen der Geschmack wichtiger als der gesundheitliche Aspekt von Lebensmitteln?	Is the taste of food more important than the quality when you evaluate food?
6	Sind Sie bereit mehr Geld für gesünderes Essen auszugeben?	Are you willing to spend more money to have healthier food?
7	Sorgt Sie der Gedanke an Ihre Ernährung mehr als 3 Stunden täglich?	Does the thought about food worry you for more than three hours a day?
*8*	*Erlauben Sie sich gegen Ihre Essprinzipien zu verstoßen*?	*Do you allow yourself any eating transgressions*?
*9*	*Glauben Sie*, *dass Ihre Stimmung Ihr Essverhalten beeinflusst*?	*Do you think your mood affects your eating behavior*?
10	Glauben Sie, dass die Überzeugung ausschließlich gesunde Lebensmittel zu essen, das Selbstwertgefühl steigert?	Do you think that the conviction to eat only healthy food increases self-esteem?
11	Glauben Sie, dass gesund zu essen Ihren Lebensstil verändert? (Häufigkeit von Restaurantbesuchen, Freizeitaktivitäten, usw.)	Do you think that eating healthy food changes your lifestyle (frequency of eating out, friends, …)?
12	Glauben Sie, dass gesundes Essen Ihr Aussehen verbessern könnte?	Do you think that consuming healthy food may improve your appearance?
*13*	*Fühlen Sie sich schuldig*, *wenn Sie gegen Ihre Essprinzipien verstoßen*?	*Do you feel guilty when transgressing*?
*14*	*Glauben Sie*, *dass es auch ungesunde Lebensmittel im Handel gibt*?	*Do you think that on the market there is also unhealthy food*?
15	Sind Sie während Ihrer Mahlzeiten alleine?	At present, are you alone when having meals?

Scoring grid for ORTO-15 test and item responses (Answer categories: Always-Often-Sometimes-Never). Scoring grid for items: 3/4/6/7/10/11/12/14/15: 1-2-3-4. Scoring grid for items 1/13: 2-4-3-1. Scoring grid for items 2/5/8/9: 4-3-2-1; items in italic were removed after statistical analysis, leaving the German version (ORTO-9-GE).

To check for clarity or spelling issues, we used a sample of 25 students to evaluate the final version of the questionnaire. Again, we included all final remarks from this evaluation into the final version of the questionnaire.

#### Self-reported eating behavior questions

We assessed self-reported dieting behavior and questions regarding weight changes. We asked participants about food intolerances, dieting frequency, dieting styles (vegan, vegetarian, mixed diet), prevalent eating disorders, prevalent mental disorders and lifetime weight changes (for detailed listing, see S1 Table).

### Statistical analyses

For statistical analyses we used several approaches. First, to determine the factor structure of the translated version of the ORTO-15, we used Confirmatory Factor Analyses (CFA). To evaluate validity and reliability check for all models, additional to Cronbach’s alpha, we applied Composite Reliability (CR), Average Variance Extracted (AVE), Maximum Shared Variance (MSV), and Average Shared Variance (ASV) [24].

In a second step, we analyzed the relationship between the mean scores for the best fitting model. Normal distribution of the continuous variables was tested by Shapiro-Wilk test. For normally distributed continuous variables, we used parametric methods (Spearman), nonparametric methods were used for non-normally distributed data (Kruskal-Wallis; Mann-Whitney). Jockheere’s test was used to test for an ordered pattern between group differences in non-normally distributed data. The Bonferroni post-hoc test was used to correct for multiple comparisons.

Besides CFA, were we used IBM SPSS 22 AMOS, all statistical analyses were carried out using IBM SPSS 22 software package.

## Results

### Confirmatory factor analysis (CFA)

In total, we analyzed four different models. Model I is based on the original three-factor structure of the ORTO-15 questionnaire (cognitive-rational, clinical, emotional). Model II is a single-factor model. Models III and IV are shortened versions of the questionnaire after omitting six items with low-item correlations and factor loadings during a model fitting process. The established ORTO-9-GE version was again evaluated without (model III) and with inter-item covariation suggested by modification indices (model IV).

#### Modeling the ORTO-15 (Model I and Model II)

**Fig 1:**
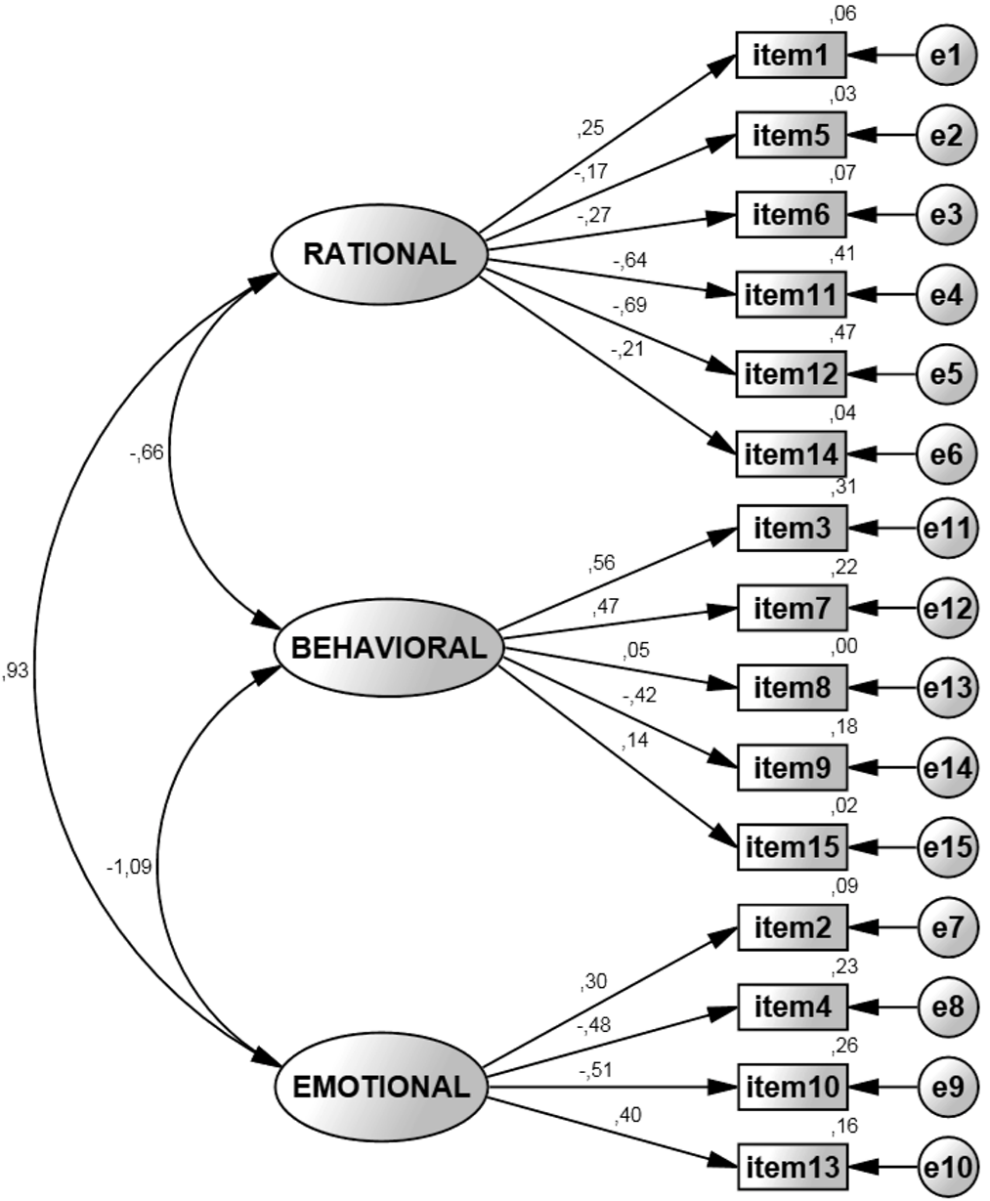
ORTO-15 as originally hypothesized by Donini and colleagues (3-factor structure). The displayed values are unstandardized regression weights from the study sample in the original 3-factor structure. Squares represent items, oval circles represent factors, squares represent questionnaire items and small circles represent error terms.

The first model tested if our data fit well with the original structure postulating a three-factor model as proposed by Donini, Marsili [5]. The CFA revealed that the three-factor solution had to be rejected due to poor model fit (χ2 = 466.38; p < .001; CMIN/DF = 5.361; CFI = .78; TLI = .74; RMSEA = .065; PCLOSE < .001; see Fig 1). Internal consistency was unacceptably low (Cronbach’s alpha = 0.303).

We tested a single-factor Model (Model II), which was rejected due to poor model fit, likewise (χ2 = 540.509; p < .001; CMIN/DF = 6.006; CFI = .74; TLI = .69; RMSEA = .070; PCLOSE < .001; see S2 Fig).

#### Modeling the ORTO-15 (Model III and Model IV)

Model I and Model II had very poor fit indices, thus we conducted an item analysis to evaluate the appropriateness of each item and improve overall model fitness. We omitted six items with low item-total correlations and low factor loadings (items: 14, 13, 9, 8, 2, 1), to improve the goodness of fit in our model (see Table 2). After item omission, CFA was conducted, but the indicators for Model III still remained inadequate (χ2 = 225.604; p < .001; CMIN/DF = 8.356; CFI = .821; TLI = .761; RMSEA = .085; PCLOSE < .001, see S3 Fig).

**Table 2:** Item analysis of the ORTO-15 questionnaire (Model 2 and Model 4).

			Original (15-item version, Model 2)			Final (9-item version, Model 4)		
	M	SD	Corrected item-total correlation	Standardized factor loadings in Model 2	Cronbach’s alpha if item deleted	Corrected item-total correlation	Standardized factor loadings in Model 4	Cronbach’s alpha if item deleted
**Item 1 (reversed)**	2.74	1.05	.11	.07	.38	-	-	-
**Item 2 (reversed)**	1.42	.63	.12	.08	.34	-	-	-
**Item 3**	3.17	.82	.15	.22	.27	.35	.34	.64
**Item 4**	3.00	.84	.24	.24	.24	.42	.39	.62
**Item 5**	2.24	.72	.19	.02	.26	.16	.15	.67
**Item 6**	1.97	.75	.25	.05	.24	.23	.26	.66
**Item 7**	3.62	.73	.16	.19	.26	.33	.37	.64
**Item 8**	2.35	.66	.10	.00	.29	-	-	-
**Item 9 (reversed)**	2.57	.83	.17	.16	.38	-	-	-
**Item 10**	2.74	.94	.32	.32	.19	.46	.62	.61
**Item 11**	2.40	.94	.32	.36	.19	.47	.67	.60
**Item 12**	2.03	.92	.29	.38	.20	.49	.66	.60
**Item 13 (reversed)**	2.41	1.08	.20	.18	.42	-	-	-
**Item 14**	1.33	.63	.10	.03	.29	-	-	-
**item 15 (reversed)**	2.83	.59	.01	.02	.31	.08	.11	.68

All 15 items of the ORTO-15 displayed with M = mean and SD = standard deviation. For model 2 and model 4, corrected item-total correlations, standardized factor loadings and reached Cronbach’s alpha values when the according item would be deleted.

**Fig 2:**
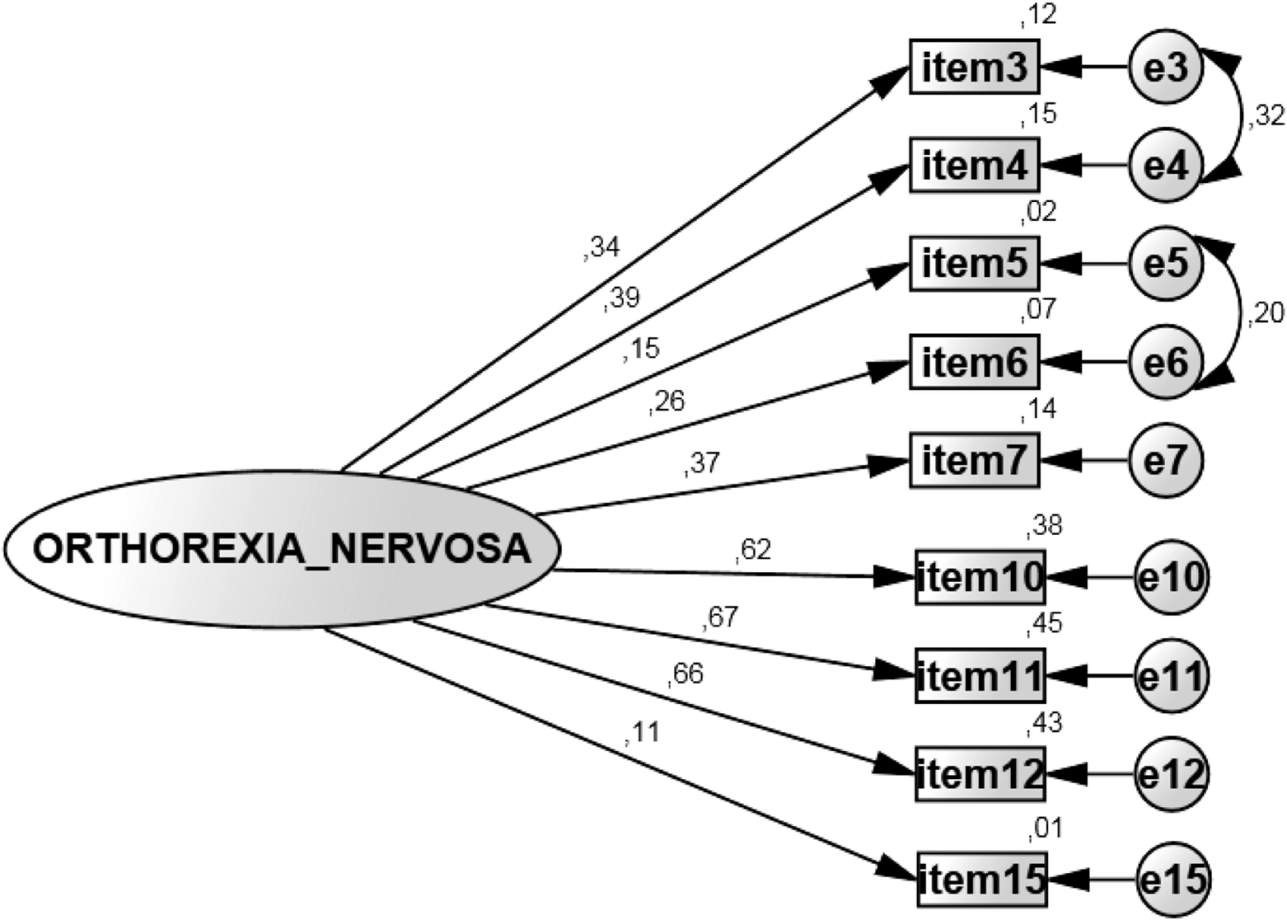
ORTO-9-GE factor structure after item omission and model fit (1-factor structure). The displayed values are unstandardized regression weights from the study sample after model fit and correlation of error terms: 3/4, 5/6. Squares represent items, oval circles represent factors, squares represent questionnaire items and small circles represent error terms.

To improve the model, variances of the error terms were analyzed through modification indices. Following the cut-off criteria of modification indices equal to or higher than 40, two error terms were correlated: 3/4 (91.712) and 5/6 (40.197). The error-term correlations were incorporated into Model IV generating a new single-factor structure with acceptable goodness of fit indices (χ2 = 83.865; p < .001; CMIN/DF = 3.355; CFI = .947; TLI = .92; RMSEA = .048; PCLOSE = .602, see Fig 2).

Internal consistency of the shortened 9-item version of the questionnaire was still low, but overall acceptable (Cronbach’s alpha = .67). The full text of the original ORTO-15 and its German version, ORTO-9-GE can be found in Table 1.

### Descriptive statistics and ON tendencies among different populations

The mean score of the ORTO-9-GE questionnaire was 24.52 (SD = 3.58), ranging from 13 to 36 points with 69.1% of the participants showing ON tendencies (cut-off <26.7). Male (24.96 ± 3.56) compared to female participants (24.36 ± 3.58) differ significantly in their ON tendencies, U = 91575, z = -2.09, p < .05, r = -.06. Weak positive correlations between ORTO-9-GE scores and age (r = .13; p < .01) and a weak negative correlation with BMI (r = -.09; p < .01). ORTO-9-GE scores did not differ significantly within education levels (compulsory school, secondary school, academic, other school), H (3) = 2.83, p = .42 and the current housing situation (living alone, with parents, in a flat share, with children other living situations) did not influence ORTO-9-GE scores, H (4) = 7.93, p = .09.

Additionally, we examined different population groups in our study sample. Our data showed that students (24.10 ± 3.39) compared to non-students (24.75 ± 3.67) had significantly lower ORTO-9-GE scores, U = 111545, z = -2.48, p < .05, r = -.07, but there was no difference between nutritional students (24.09 ± 3.26) students from other disciplines (24.12 ± 3.55), H (1) = .41, p = .52. Flight attendants (24.98 ± 3.38) compared to other professions (24.48 ± 3.59) did not differ in ORTO-9-GE, U = 30230, z = -1.21, p = .22, r = -.006 and there were no differences between health professionals (24.67 ± 3.40) and individuals without health profession background (24.49 ± 3.62) in orthorectic tendencies, U = 66966, z = -.64, p = .52, r = -.01 (see S2 Table).

#### Self-reported eating behavior and ON tendencies

Individuals who report to follow a very strict eating schedule (23.65 ± 3.71) compared to those who report not to follow a strict eating schedule (24.62 ± 3.50) showed lower ORTO-9-GE scores, U = 50664, z = -2.97, p < .01, r = -.09. Individuals who are convinced to exclusively eat healthy foods (23.37 ± 4.26) compared to those who do not agree with this statement (24.63 ± 3.40) showed significantly lower ORTO-9-GE scores, U = 41581, z = -3.58, p < .01, r = -.11. In addition, individuals who report to spend a large amount of time with the preparation of meals (23.85 ± 3.56) compared to those who not spend a large amount of time for meal preparation (25.18 ± 3.40) showed significantly lower ORTO-9-GE scores, U = 91477, z = -5.48, p < .01, r = -.17. ORTO-9-GE scores in individuals who report to be reluctant to eat food that is prepared by others (22.00 ± 3.97) compared to those who do not agree with this statement (24.79 ± 3.36), differed significantly, U = 27631, z = -7.03, p < .01, r = -.23 (see Table 3).

**Table 3:** Associatons between self-reported health and eating behaviors and ORTO-9-GE scores.

	**Agreement**	**Diasagreement**	**Statistics**	**Significance**
I don’t like to eat foods prepared by others.	22.00 ± 3.97	24.79 ± 3.36	z = -7.025	< 0.001
I consume only healthy foods.	23.37 ± 4.25	24.63 ± 3.41	z = -3.588	< 0.001
I always eat according to my eating schedule.	23.65 ± 3.71	24.62 ± 3.50	z = -2.970	< 0.05
	**Frequency**	**ORTO-9-GE scores (M** ± **SD)**	**Statistics**	**Significance**
Food intolerance	≤ 1 food intolerances	24.54 ± 3.59	z = -2.395	< 0.05
	> 2 intolerances	22.11 ± 1.96		
Eating disorder	current	19.95 ± 3.29	z = -5.39	< 0.001
	no eating disorder	24.62 ± 3.53		
Mental disorders (obsessive-compulsive disorder, depression, anxiety disorder)	current	22.67 ± 4.39	z = -2.66	< 0.001
	no mental disorder	24.59 ± 3.54		

Self-reported eating and health behaviors and ORTO-9-GE scores. M = mean; SD = standard deviation; test statistic for non-parametric tests (z-scores and p-values indicated).

**Fig 3:**
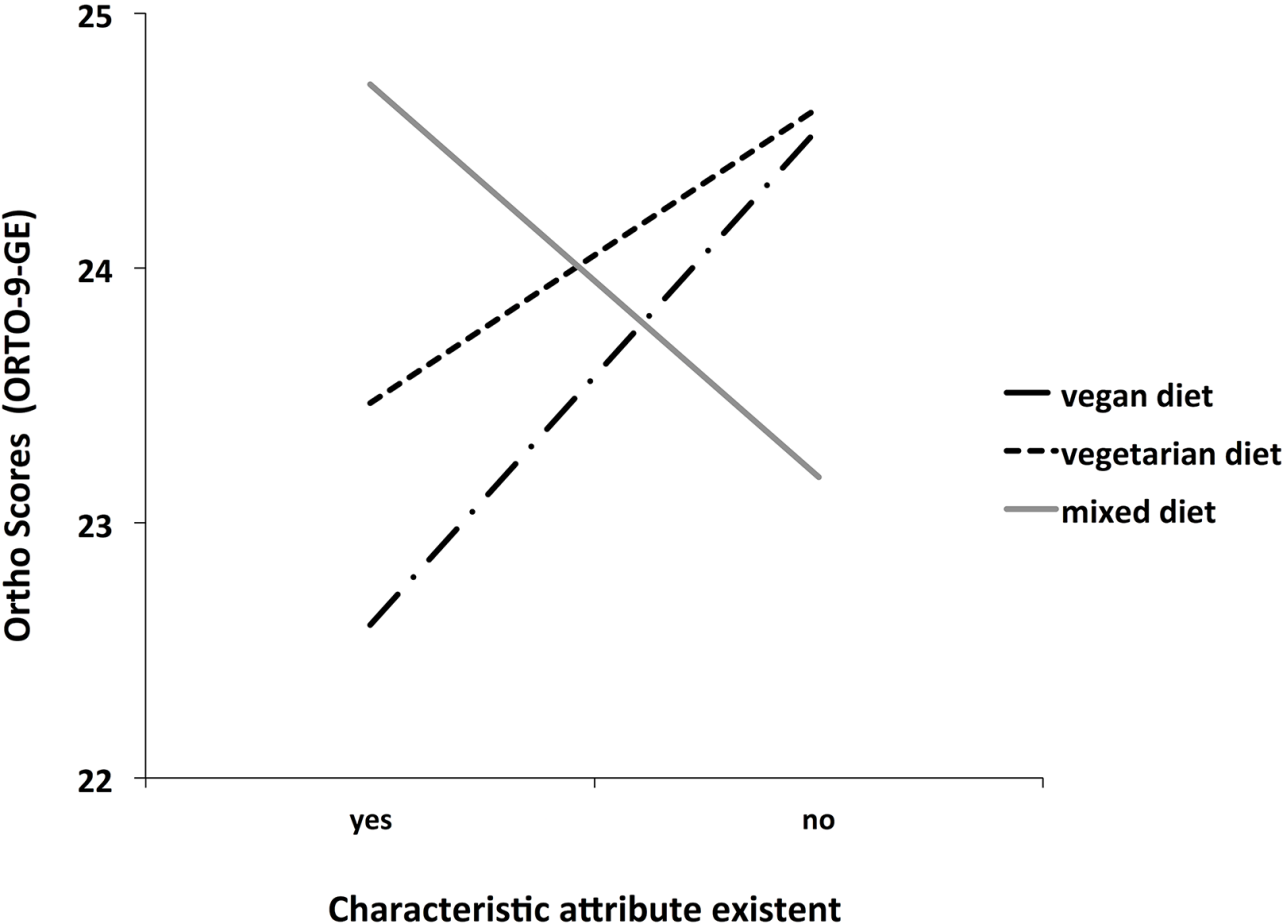
ORTO-9-GE scores associated with dieting style. ORTO-9-GE scores as a function of dieting style. Participants dieting style defined as either vegan, vegetarian or having a mixed diet. Lower ORTO-9-GE scores indicate higher orthorectic tendencies.

Self-reported intolerances also influenced ON scores. Individuals who indicate to have 2 or more food intolerances (22.11 ± 1.96) compared to those who report one or no self-reported food intolerances (24.54 ± 3.59) showed siginificantly higher ON tendencies, U = 2471, z = -2.39, p < .05, r = -.07. Individual dieting style is also associated with ON scores. Individuals who are on a vegetarian (23.47 ± 3.64) or a vegan diet (22.6 ± 3.82) show higher ON tendencies than individuals on a mixed diet (24.72 ± 3.47), H (1) = 22.16, p < .01 (see Fig 3).

**Fig 4:**
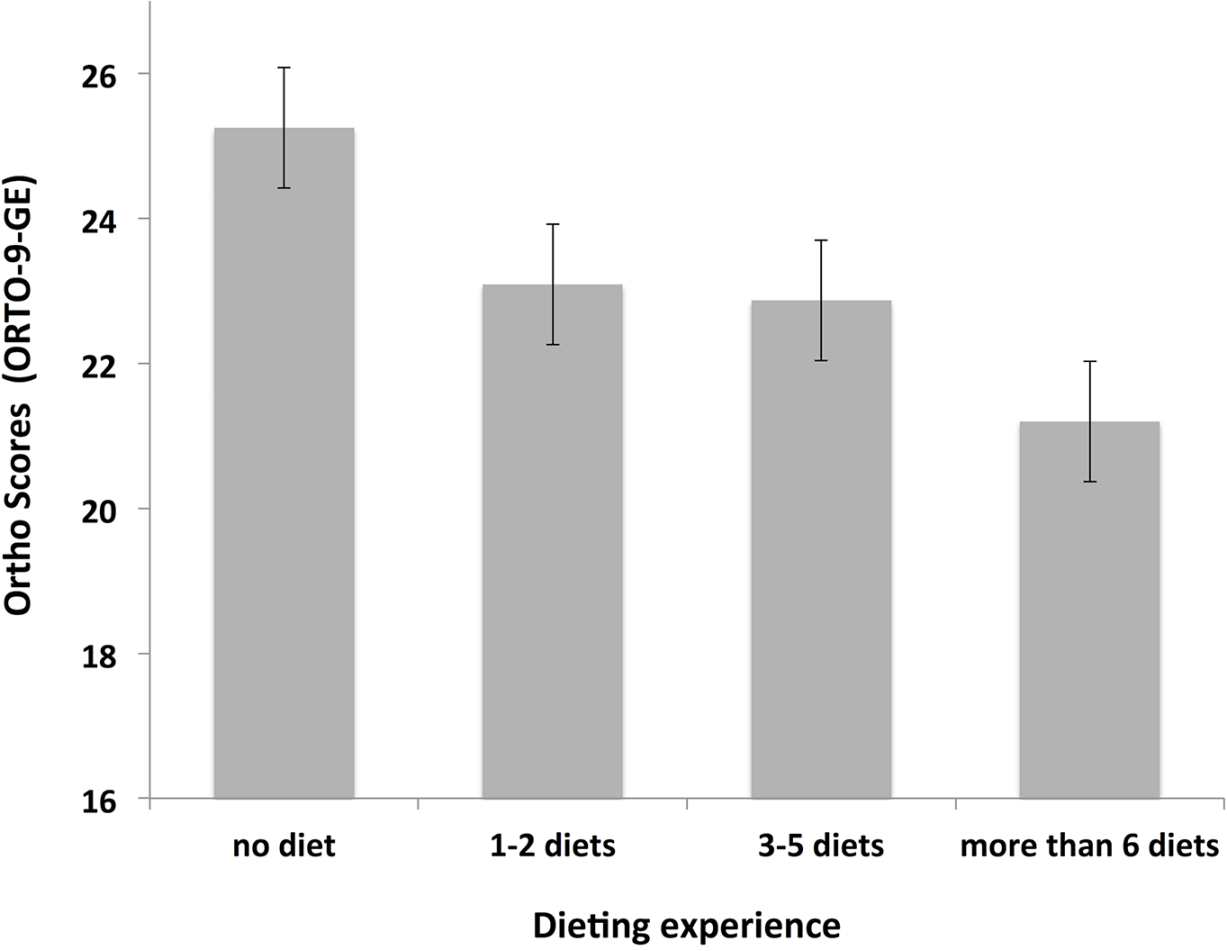
ORTO-9-GE scores associated with dieting experience. ORTO-9-GE scores as a function of dieting experience (range: no dieting experience to > 6 diets). Lower ORTO-9-GE scores indicate higher orthorectic tendencies. Error bars indicate standard errors of the means.

Dieting experience affects ORTO-9-GE scores. Higher ON tendencies are found among individuals with profound dieting experience; individuals with no dieting experience showed increased ORTO-9-GE scores (25.25 ± 3.31) compared to individuals with 1–2 diets (23.09 ± 3.71), 3–5 diets (22.87 ± 3.55) and more than 6 diets (21.20 ± 3.56), H (1) = 57.78, p < .01 (see Fig 4).

#### Self-reported eating, mental disorders and weight change and ON tendencies

Current self-reported eating disorders also influenced ORTO-9-GE scores: individuals who report a current eating disorder showed higher ON tendencies (19.95 ± 3.28) compared to those not reporting an eating disorder (24.61 ± 3.52), U = 3668, z = -5.39, p < .01, r = -.16. Hence, individuals who indicate a current mental disorder (obsessive-compulsive disorder, depression, anxiety disorders) showed lower ORTO-9-GE scores (22.67 ± 4.39) compared to those without any of these conditions (24.59 ± 3.54), U = 13227, z = -2.63, p < .01, r = -.08.

**Fig 5:**
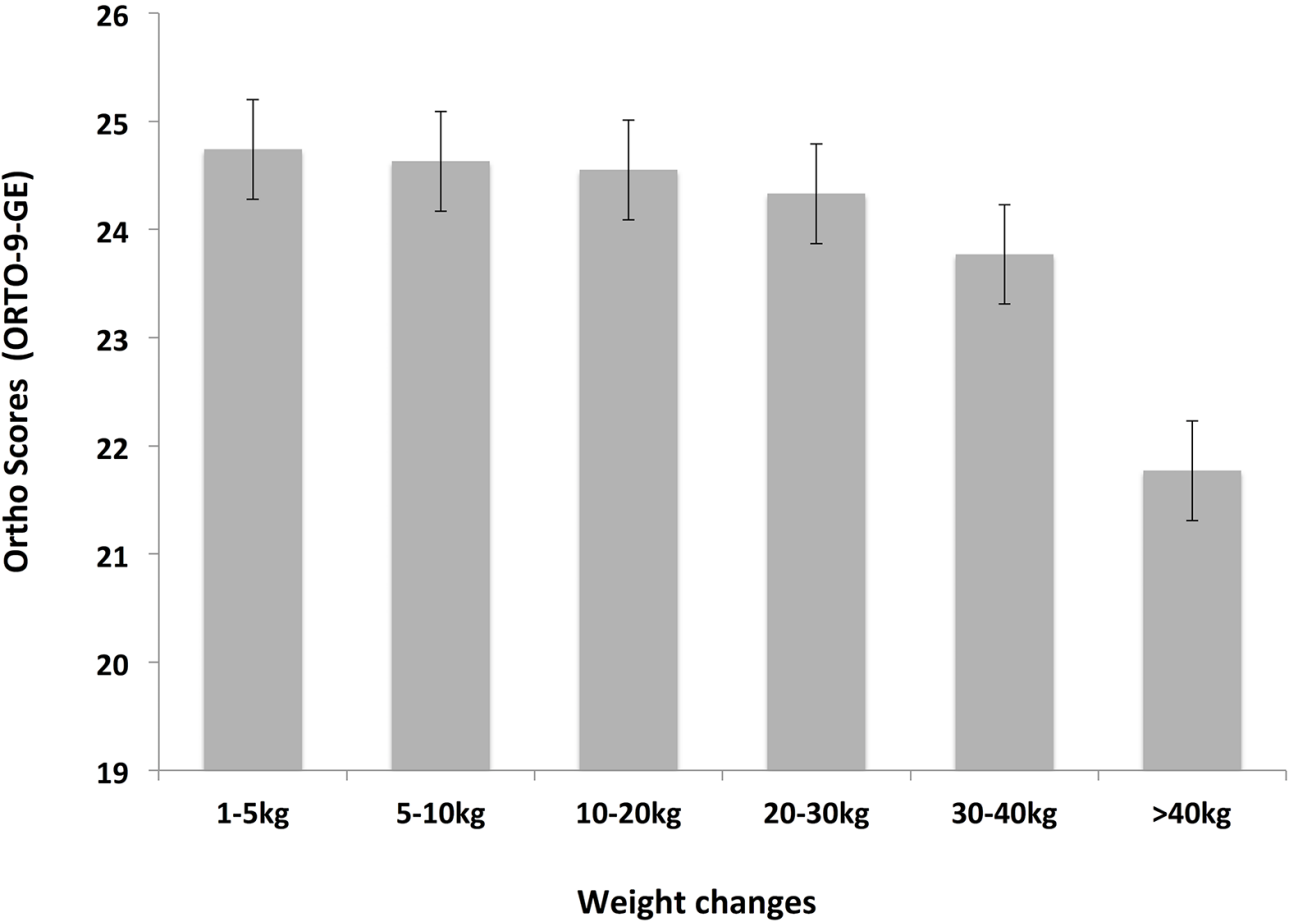
ORTO-9-GE scores associated with lifetime weight changes. ORTO-9-GE scores as a function of lifetime weight changes (range from 1-5kg to > 40kg lifetime weight change). Lower ORTO-9-GE scores indicate higher orthorectic tendencies. Error bars indicate standard errors of the means.

Our data showed that past weight changes influenced ON tendency. Individuals who report major lifetime weight changes (>40 kg), showed significantly lower ORTO-9-GE scores (21.77 ± 3.55) compared to individuals who report only minor weight changes (5-10kg; 24.63 ± 3.46), H (1) = 19.68, p < .01. Jockheere’s test revealed a significant trend in the data: as self-reported weight change increased, ORTO-9-GE scores decreased, J = 4071, z = -4.43, p < .01, r = .14 (see Fig 5).

## Discussion

The present study aimed to investigate the psychometric properties of a translated German version of the ORTO-15 instrument among a heterogeneous population sample. So far only a few studies have investigated the psychometric properties of the ORTO-15 questionnaire. This study was the first attempt to validate the original version translated into German language. Our data showed conflicting as well as overlapping results compared with results from previous studies.

We found that eating behavior traits which deviate from eating behavior considered as normal, such as strict eating schedules and the reluctance to eat food prepared by others are associated with higher ON tendencies. This finding is at the core of ON and in accordance with previous studies [10]. To our knowledge, this is the first study to demonstrate that individuals with ON tendencies are more likely to be on a vegetarian or vegan diet. This is only moderately surprising, because being on a vegetarian diet requires a fair degree of self-discipline, planning and cognitive processing related to eating behavior. Besides ethical considerations, health aspects have been identified as the main motive for becoming vegan or vegetarian [25]. However, a recent study by Burkert, Muckenhuber [26] could show that poorer overall health condition (e.g. allergies, mental health disorders), a higher need for health care and poorer quality of life is associated with a vegetarian diet. Albeit only reported anecdotally, ON is associated with a shortage of essential nutrients and malnutrition due to an unbalanced diet [4, 19]. In our study, individuals who reported ≥ 2 food intolerances showed higher ON tendencies. Together, the results of this study can be seen as a reference that preceding ON tendencies in individuals on a vegetarian or vegan diet may be a behavioral manifestation and a possible explanation for having poorer overall health condition.

Additionally, our data support the idea that more frequent dieting experiences increase ON tendencies reflecting previous research results [10, 18, 21, 27]. Nutrition students did not differ in their ON tendencies compared to students from other disciplines. This finding is intriguing regarding the assumption that nutritional students are more prone for dieting behavior than their counterparts from other disciplines. Another pivotal outcome from this study is, that major lifetime weight changes (> 40 kg) and those individuals with currently diagnosed eating disorders showed an increased tendency for ON. Past or recent eating disorders are mostly accompanied by major weight fluctuations [28] and in either direction of weight change (under/overweight), an adaptation of eating behavior may be relevant for treatment. Therefore, an explanation for decreased ON scores may be given.

### Limitations and generic problems with the ORTO-15 measure

Some limitations of the present study should be taken into account. The presented data analyses rely on self-reported data via online assessemt. Self-reported weight and height is highly biased as shown by previous studies (e.g. underreporting) [29]. Recall bias can not be excluded in the present study. Secondly, behavioral data on eating behavior was measured indirectly and is not based on experimental or observational data. Interpretation of self-reported behavior (e.g having a strict eating schedule) should be cautious.

### Generic problems of the ORTO-15 measure

Our findings show that after a thorough translation process of the original version of the ORTO-15 questionnaire, an instrument with only mediocre validity could be constructed (ORTO-9-GE). After CFA, the best fitting model showed weak psychometric properties and it was necessary to omit six items from the original 15-item questionnaire (omitting 40%) to reach moderately acceptable construct validity. Sufficient, but still only moderate internal reliability points to the fact that the original questionnaire may be flawed from scratch. This finding is not newly established, since even Donini who is the author of the original questionnaire admitted the psychometric flaws of the original version of the ORTO-15 [5]. This fact has been widely ignored by the scientific community and rather than developing new and better tools, no alternatives for the ORTO-15 have been constructed so far. For instance, item 1 of the ORTO-15 seems to be problematic: *When eating*, *do you pay attention to the calories of the food*? The scoring grid for this question is ambiguous (always and never score low, often and sometimes score high) as well as the intention of the question. Besides calories, eating exclusively healthy food is not always associated with calories-only, but other beliefs about foods are present. As such, in a case study Moroze, Dunn [7] reported that almost mystic beliefs about magical properties of broccoli or a conviction about healthy properties of certain micronutrients are present in individuals with ON.

Our present findings may explain the incongruent and often contradictive findings from studies using the ORTO-15 instrument and adapted versions. Our study suggests that the internal validity of the original ORTO-15, without undergoing an extensive adaptation process and model fit, would be of insufficient internal reliability (Cronbach’s alpha = .30). Large-scale investigations of ON are mainly based on the ORTO-15 measure, but in most studies validity and reliability (Cronbach’s alpha) was of poor construct validity [19, 21, 30, 31] or were not reported [5, 16, 32, 33]. Only few studies reported acceptable construct validity [10, 34]. On the other hand it has been argued that the usage of Cronbach’s alpha is not a reliable tool for assessing the validity of an instrument [35]. Still, to reach acceptable validity with incremental fit indices [36], we had to delete almost half of the original items. Varga, Thege [10] argue that the inconsistency of the assessment may be partly due to cultural differences between countries. This argument may be very speculative and is not supported by data from relevant studies. Our research conflicts with this cultural-differences argument and indicates that cultural differences may be less of a problem, but rather the original construction of the ORTO-15 being the more fundamental problem. First, Austria is a heterogeneous country with heterogeneous cultural backgrounds among citizens (similar to Germany and Switzerland). A general definition of an instrument to measure behavioral traits should measure reliably across different population groups, not solely provide valid data among participants from e.g. Western, Educated, Industrialized, Rich, and Democratic (WEIRD) study samples [37]. Using different instruments depending on cultural or religious backgrounds may be unrewarding on the one and not applicable on the other hand. Second, a solid measure should take basal mechanisms describing the pathology of ON into account. When omitting 40% of the instrument, important information of the original instrument may get lost and results ultimately become vague. As shown in our study, to increase the reliability of the questionnaire, we had to omit relevant questions regarding ON pathology (items: 14, 13, 9, 8, 2, 1); for instance, the information whether or not individuals feel guilty when transgressing their food habits (item 13) is of particular importance for ON. The statistical necessity to omit this item can hardly be argued with cultural differences, but rather is a result of the overall construction problem of the original ORTO-15 questionnaire. This should be considered in futures studies investigating ON.

### Conclusion

In conclusion, ON is framed by a variety of factors due to its complex nature. Our research shows that several eating and dieting behaviors influence ON tendencies. Most importantly, this study indicates that the ORTO-15 measurement entails some basic psychometric flaws and its usage should be rethought from scratch.

## Supporting Information

S1 Fig Flow Chart of the study. (PDF)

S2 Fig ORTO-15 factor structure (1-factor structure). The displayed values are unstandardized regression weights from the study sample in a 1-factor structure. Squares represent items, oval circles represent factors, squares represent questionnaire items and small circles represent error terms.
(TIFF)

S3 Fig ORTO-9-GE factor structure after item omission (1-factor structure). The displayed values are unstandardized regression weights from the study sample in a 1-factor structure after omitting item: 1/2/8/9/13/14. Squares represent items, oval circles represent factors, squares represent questionnaire items and small circles represent error terms.
(TIFF)

S1 Table Lifestyle and eating behavior questions. (DOCX)

S2 Table Associations between ORTO-9-GE scores and descriptives of the study population. The table shows descriptives of the study sample and according ORTO-9-GE scores. M = mean; SD = standard deviation.
(DOCX)
