# Supplementary material for: When Eating Right, Is Measured Wrong! A Validation and Critical Examination of the ORTO-15 Questionnaire in German
Source: PLoS One. 2015 Aug 17;10(8):e0135772. doi: 10.1371/journal.pone.0135772 (PMC4539204; doi:10.1371/journal.pone.0135772)
Supplement: S1 Fig — (PDF) [file pone.0135772.s001.pdf]

PRISMA 2009 Flow Diagram

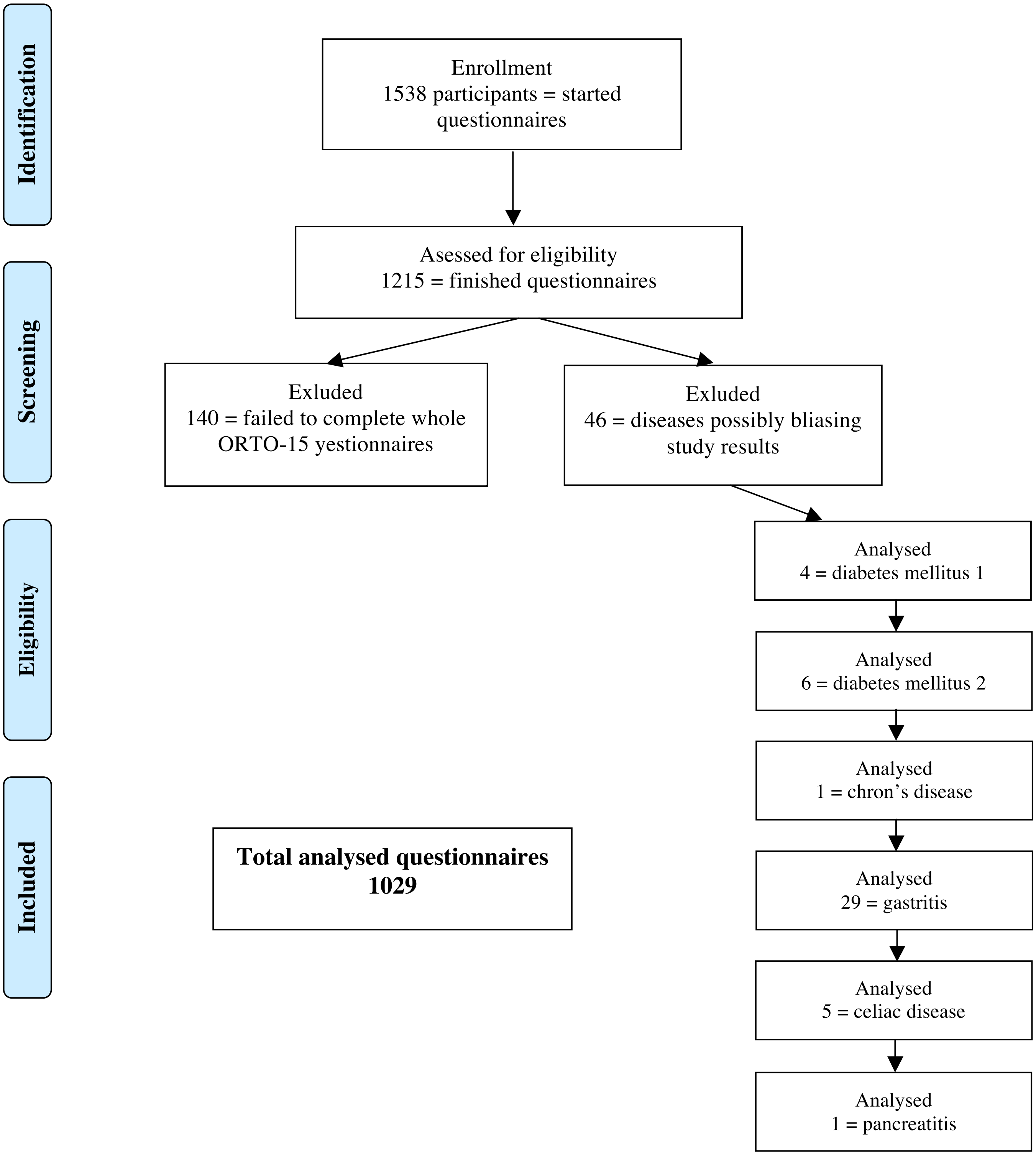

From: Moher D, Liberati A, Tetzlaff J, Altman DG, The PRISMA Group (2009). *P*referred *R*eporting *I*tems for *S*ystematic Reviews and *M*eta-*A*nalyses: The PRISMA Statement. PLoS Med 6(6): e1000097. doi:10.1371/journal.pmed1000097

For more information, visit www.prisma-statement.org.
