# Supplementary figures and images for: When Eating Right, Is Measured Wrong! A Validation and Critical Examination of the ORTO-15 Questionnaire in German
Source: PLoS One. 2015 Aug 17;10(8):e0135772. doi: 10.1371/journal.pone.0135772 (PMC4539204; doi:10.1371/journal.pone.0135772)

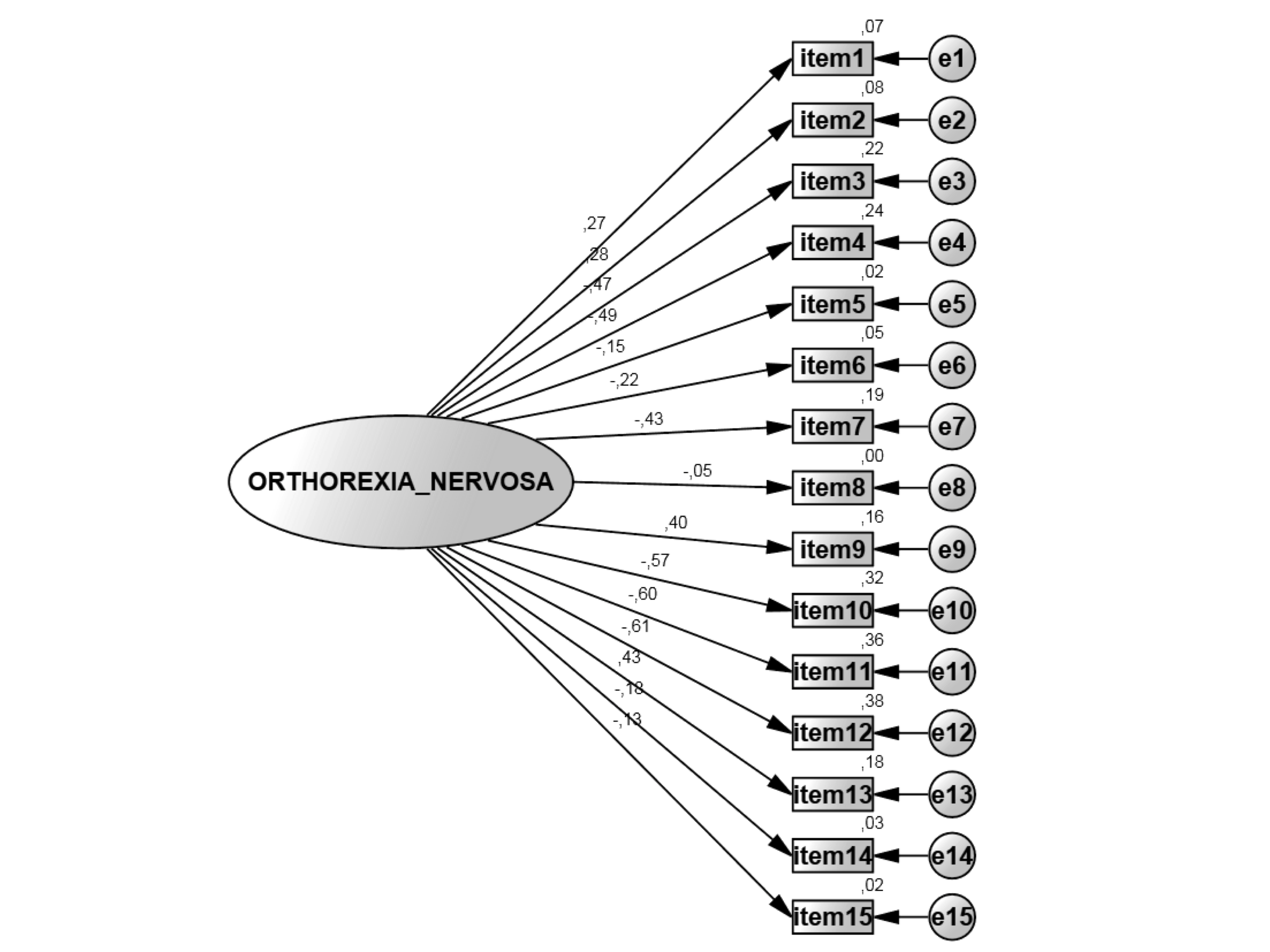

Supplement: S2 Fig — The displayed values are unstandardized regression weights from the study sample in a 1-factor structure. Squares represent items, oval circles represent factors, squares represent questionnaire items and small circles represent error terms. (TIFF) [file pone.0135772.s002.tiff]

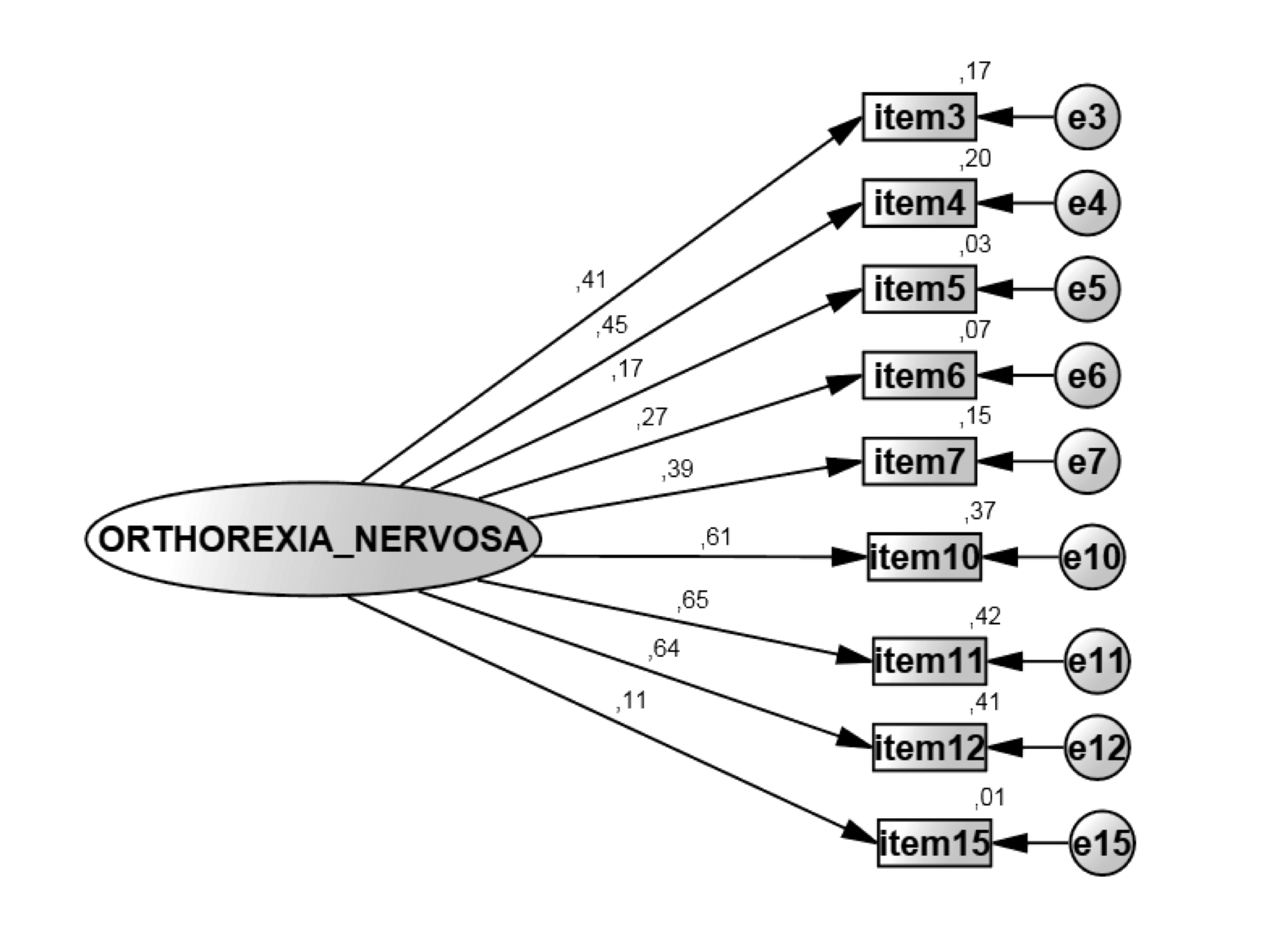

Supplement: S3 Fig — The displayed values are unstandardized regression weights from the study sample in a 1-factor structure after omitting item: 1/2/8/9/13/14. Squares represent items, oval circles represent factors, squares represent questionnaire items and small circles represent error terms. (TIFF) [file pone.0135772.s003.tiff]
