# Supplementary material for: When Eating Right, Is Measured Wrong! A Validation and Critical Examination of the ORTO-15 Questionnaire in German
Source: PLoS One. 2015 Aug 17;10(8):e0135772. doi: 10.1371/journal.pone.0135772 (PMC4539204; doi:10.1371/journal.pone.0135772)
Supplement: S1 Table — (DOCX) [file pone.0135772.s004.docx]

| **Topic** | **Statement/question** | **Modus** |
| --- | --- | --- |
| **Eating behavior** |  |  |
|  | I always eat according to my eating schedule. | Binominal (yes/no) |
|  | I consume only healthy foods. | Binominal (yes/no) |
|  | I spend a large amount of time preparing my meals. | Binominal (yes/no) |
|  | I don’t like to eat food that were prepared by others. | Binominal (yes/no) |
| **Lifetime weight changes** | **Indicate your weight** |  |
|  | Indicate your highest weight | Binominal (yes/no) |
|  | Indicate your lowest weight | Binominal (yes/no) |
| **Food Intolerances** | **Indicate your food intolerances** |  |
|  | Histamin | Binominal (yes/no) |
|  | Lactose | Binominal (yes/no) |
|  | Fructose | Binominal (yes/no) |
|  | Others not listed | Straight answer |
| **Dieting style** | **Indicate your dieting style** |  |
|  | Vegan | Binominal (yes/no) |
|  | Vegetarian | Binominal (yes/no) |
|  | Mixed diet | Binominal (yes/no) |
| **Dieting experience** | **Indicate your experience with following diets** |  |
|  | Clean Eating | Binominal (yes/no) |
|  | Dukan Diet | Binominal (yes/no) |
|  | Paleo | Binominal (yes/no) |
|  | Atkins Diet | Binominal (yes/no) |
|  | My Line | Binominal (yes/no) |
|  | 10 in 2 Diet | Binominal (yes/no) |
|  | Logi-method | Binominal (yes/no) |
|  | Food combining diet | Binominal (yes/no) |
|  | Raw-foodism | Binominal (yes/no) |
|  | Low Carb | Binominal (yes/no) |
|  | Weight Watchers | Binominal (yes/no) |
|  | Metabolic Balance | Binominal (yes/no) |
|  | Others not listed | Straight answer |
| **Mental Disorders** | **Indicate current mental disorders** |  |
|  | obsessive-compulsive disorder | Binominal (yes/no) |
|  | depression | Binominal (yes/no) |
|  | anxiety disorder | Binominal (yes/no) |
| **Eating disorders** | **Indicate current eating disorders** |  |
|  | Anorexia Nervosa | Binominal (yes/no) |
|  | Bulimia Nervosa | Binominal (yes/no) |
|  | Binge Eating Disorders | Binominal (yes/no) |
|  | Night Eating Syndrome | Binominal (yes/no) |
