# Supplementary material for: When Eating Right, Is Measured Wrong! A Validation and Critical Examination of the ORTO-15 Questionnaire in German
Source: PLoS One. 2015 Aug 17;10(8):e0135772. doi: 10.1371/journal.pone.0135772 (PMC4539204; doi:10.1371/journal.pone.0135772)
Supplement: S2 Table — The table shows descriptives of the study sample and according ORTO-9-GE scores. M = mean; SD = standard deviation. (DOCX) [file pone.0135772.s005.docx]

| **Decriptives** | **Frequency** | **ORTO-9-GE scores (M ± SD)** | **Statistics** | **Significance** |
| --- | --- | --- | --- | --- |
| **Education level** | Compulsory School | 24.51 ± 4.27 | χ^2^ = 2.83 | 0.42 |
|  | Secondary School | 24.44 ± 3.65 |  |  |
|  | Academic | 24.65 ± 3.40 |  |  |
|  | Other | 23.76 ± 3.85 |  |  |
| **Population groups** | Men  Women  Health professionals  Others  Flight attendants  No flight attendants  Students  No students  Students nutrition  Other students  No students | 24.96 ± 3.56  24.36 ± 3.58  24.67 ± 3.40  24.49 ± 3.62  24.98 ± 3.38  24.48 ± 3. 59  24.11 ± 3.39  24.76 ± 3.67  24.10 ± 3.26  24.12 ± 3.55  24.76 ± 3.67 | Z = -2.01  Z = -0.64  Z = -1.22  Z = -2.48  χ^2^ = 0.41 | 0.04  0.52  0.22  < 0.05  0.52 |
| **Housing situation** | Living alone  Living with parents  Living in a flat share  Living with children  Other living situations | 24.17 ± 3.66  24.40 ± 3.63  24.46 ± 3.50  24.96 ± 3.66  25.28 ± 4.04 | χ^2^ = 7.93 | 0.09 |
